# Prescription and Effects of Cardiorespiratory Training in Individuals with Intellectual Disability: A Systematic Review

**DOI:** 10.3390/healthcare11142106

**Published:** 2023-07-24

**Authors:** Miguel Jacinto, Rafael Oliveira, Alexandre D. Martins, João Paulo Brito, Rui Matos, José Pedro Ferreira

**Affiliations:** 1 Faculty of Sport Sciences and Physical Education, University of Coimbra, 3040-248 Coimbra, Portugal; jpferreira@fcdef.uc.pt; 2 ESECS, Polytechnic of Leiria, 2411-901 Leiria, Portugal; rui.matos@ipleiria.pt; 3 Life Quality Research Centre (CIEQV), 2040-413 Rio Maior, Portugal; rafaeloliveira@esdrm.ipsantarem.pt (R.O.); alexandremartins@esdrm.ipsantarem.pt (A.D.M.); jbrito@esdrm.ipsantarem.pt (J.P.B.); 4 Sports Science School of Rio Maior, Polytechnic Institute of Santarém, 2040-413 Rio Maior, Portugal; 5 Research Center in Sport Sciences, Health Sciences and Human Development (CIDESD), 5001-801 Vila Real, Portugal; 6 Comprehensive Health Research Centre (CHRC), Departamento de Desporto e Saúde e Desenvolvimento Humano, Universidade de Évora, Largo dos Colegiais, 7000-727 Évora, Portugal; 7 Research Center for Sport and Physical Activity (CIDAF), 3040-248 Coimbra, Portugal

**Keywords:** cardiorespiratory function, cardiorespiratory protocols, intellectual disabilities, training programs

## Abstract

This study aims to systematize effects of cardiorespiratory training (CT) programs in individuals with intellectual disability (ID) and identifying the fundamental and structuring aspects for the prescription of CT. This systematic review was carried out through four databases (Pubmed, Web of Science, Scopus, and SPORTDiscus), considering data from the period between 2013 and 2022. From 257 studies, 12 studies were included in this systematic review. Three studies used interval CT, while seven used continuous CT. Seven were carried out in the population with Down syndrome, while only three were carried out with participants with ID. The CT programs had the following characteristics: duration of 8 to 12 weeks, weekly frequency of three sessions, for 20 to 60 min, the intensity of 50% to 80% of maximal heart rate or 70% to 80% of peak oxygen consumption, using an ergometer cycle or an outdoor walking. The studies reported improvements in cardiorespiratory function, lipid, hemodynamic and metabolic profile, body composition, and neuromuscular and cognitive capacity. This review presents characteristics and recommendations that technicians can follow when structuring, prescribing, and implementing CT programs to individuals with ID.

## 1. Introduction

Persons with intellectual disability (ID) are characterized by a deficit of intellectual and adaptive functioning in the conceptual, social, and practical domain [1]. This population is currently considered a social group that demands special attention [2,3] due to their low average life expectancy correlated with the degree of ID [4,5]. Even so, there has been an increase in the average life expectancy of this population over the years [6].

Greater longevity is associated with an increase in comorbidities and health care costs in order to provide adequate care to adults with ID, in particular when they live longer than their parents [7]. The premature aging of individuals with ID starts around the fifth decade of life [8] and also represents serious health concerns [9]. Those health problems are partially attributed to their sedentary lifestyle behaviors, physical inactivity, and the impaired physical fitness associated with several factors such as possible lack of motivation, task understanding, and an unhealthy diet [10]. The sedentary lifestyle and lower rates of physical activity [11] lead to an increase in several comorbidities, such as diabetes, hypertension, and dyslipidemia, among others, when compared to persons without ID [12,13,14,15].

In addition, the low physical fitness of individuals with ID becomes clear. Low cardiorespiratory function has been associated with increases in body mass, body mass index (BMI), percentage of fat mass, and waist and hip circumferences [16,17]. Also, some studies reported that individuals with ID have lower values of maximum heart rate (HR_max_) and maximum oxygen consumption (VO_2max_) than the general population [18,19], which are directly linked to lower cardiorespiratory and ventilatory capacity [20]. Low cardiorespiratory capacity, besides negatively influencing their success in performing activities of daily living and independence, also affects their quality of life [21,22,23]. In addition, they can lead to an increase in healthcare costs and mortality rate for individuals with ID, higher healthcare resource utilization such as outpatient office visits, inpatient hospitalizations, and emergency room use, and prescription drug use is higher as compared to individuals without ID [15,24,25].

Several factors may justify the physical inactivity of this population and the prevalence of sedentary lifestyles, namely the existence of barriers to the practice of physical activity (personal, family, social, financial, and environmental) [26].

Physical exercise has been shown to be an effective tool to promote all the variables previously mentioned. In one of the most cited systematic reviews, Bartlo and Klein [27] report moderate to strong evidence that physical activity positively affected balance, muscle strength, and quality of life in individuals with ID, but call for further research. Strength training has also been shown to be effective in increasing strength, balance, and lean mass while decreasing fat mass and waist circumference. As far as cardiorespiratory training (CT) is concerned, the evidence is unclear, lacks robustness in the methodology used, or focuses on only one variable. For instance, the study by Obrusnikova et al. [28] showed a positive and significant effect (SMD = 0.42, 95% CI: 0.05 to 0.79, *p* = 0.02) on increasing cardiorespiratory fitness. Some other studies with CT intervention programs, in addition to the cardiorespiratory function, have reported positive effects on muscle endurance, with a small positive effect on body composition and flexibility [24,25,29,30,31]. Based on published research, it seems that cardiorespiratory training could help to improve cardiorespiratory endurance, muscle endurance, flexibility, and body composition in individuals with ID [19].

Considering that some individuals with ID have not had the opportunity to participate in strength training sessions due to financial costs or comorbidities [26], CT can be a good alternative. In this sense, it is needful to identify the characteristics and structure of the CT programs such as intensity, type of exercise, duration, frequency, and progression in order to increase health-related outcomes in these individuals. For this purpose, we analyze studies that have provided information about the prescription of CT and its effects on the health-related parameters of adults with ID. An in-depth description of the proposed CT programs performed with people with ID can provide useful data that could help fitness and rehabilitation professionals to develop better practice guidelines and interventions.

Therefore, the main objective of the present review is to answer the two following research questions: (i) What are the effects of CT programs intervention in individuals with ID? (ii) What are the most common and effective characteristics of the intervention CT programs? This study follows a previous study on strength intervention, and it is expected to draw relevant conclusions that allow practical, evidence-based exercise recommendations to be made for maximizing the optimal CT response to exercise.

## 2. Methodology

This systematic review was carried out following the items of the Preferred Reporting Items for Systematic Reviews and Meta-Analyses (PRISMA) guidelines [32] and was carried out in the period from January 2023 to June 2023 The protocol was registered in the PROSPERO, with the number CRD42021286402 (22 November 2021). The PICOS strategy [33,34] was defined as follows: (i) “P” (patients) corresponded to participants with ID, of all ages, regardless of gender, race, or ethnicity; (ii) “I” (intervention) corresponded to any CT program performed with ID (including Down syndrome), regardless of the intervention time; (iii) “C” (comparison) corresponded to the comparison between the control group vs. the intervention group or pre and post-intervention; (iv) “O” (outcome) corresponded to CT as the first or second variable under study; (v) “S” (study design) corresponded to randomized controlled clinical trials.

### 2.1. Eligibility Criteria

Studies that provided information regarding the effects of CT interventions on the health-related and physical fitness outcomes of individuals with ID were considered eligible if they met the following inclusion criteria: (i) randomized controlled studies; (ii) intervention studies in any type of CT (e.g., continuous or interval) and with any duration (e.g., 8 weeks, 12 weeks, 24 weeks, etc.); (iii) population with ID, in different degrees (e.g., mild, moderate, severe, or profound); (iv) studies with individuals of any race, ethnicity, gender, or age group (since the American College of Sports Medicine (ACSM) also makes no distinction); (v) studies with any number of participants. In turn, all studies with the following characteristics were excluded if: (i) were published before 2013 (considering the first time that the ACSM published a chapter on guidelines for exercise testing and prescription for individuals with ID and Down syndrome in this year [35]); (ii) the research was not written in English; (iii) articles with participants with another type of disability or pathologies (e.g., multiple disabilities); (iv) articles that do not describe the intervention protocol, namely the prescription of CT in the PE program; (v) articles in which the intervention is focused on a sport modality (e.g., soccer); (vi) articles in which the intervention combines several physical abilities (e.g., CT with strength training; CT with nutrition, etc.) (since one intervention may influence the prescription of the other intervention and/or the results may not be caused by the CT); and (vii) articles in which the intervention is not just CT in the same group (example: combined training, CT and nutrition, among others).

### 2.2. Information Sources and Research Strategies

Electronic searches were carried out in the PubMed (title and abstract), Web of Science, Scopus, and SPORTDiscus (tittle/abstract/keywords), encompassing articles published between January 2013 and 9 June 2023 thus encompassing only recent literature and current evidence. At the same time, the period of retreat (2013) of the literature search coincides with the first time that the American College of Sports Medicine—ACSM published a chapter on guidelines for exercise testing and prescription for individuals with ID and Down syndrome [35]. Some medical subject headings (MeSH) descriptors and natural language [36] that we consider to complement the research were used, namely: “aerobic exercise”, “aerobic training”, “cardio training”, “cardiorrespiratory training”, “cardiorespiratory training”, “cardio exercise”, “cardiorrespiratory exercise”, “cardiorespiratory exercise”, “continuous exercise”, “continuous training”, “high-intensity interval training”, “HIIT”, “interval training”, “interval exercise”, “mental retardation”, “intellectual disability”, “intellectual disabilities”, “intellectual and developmental disabilities”, and “Down syndrome”, as indicated in Table 1.

### 2.3. Selection and Data Collection Process

The aim was to search for intervention studies, based on CT program, regardless of its purpose. The research was carried out autonomously by two authors (MJ and RO) and, after excluding duplicate articles, reading the titles and abstracts, according to the eligibility criteria, the results of both were compared and discussed. When differences arose between these two authors, a third author (JB) was available to collaborate and make a final decision. One of the authors (MJ) downloaded the main information from the articles, namely: authors’ names, year of publication, country, aims, participants, type of study, assessment instruments, duration/frequency, exercises, intensities, and main results.

### 2.4. Methodological Quality

The quality assessment of each study was performed based on the PEDro scale and its database, from the Physiotherapy Evidence Database [37,38]. The scale consists of 11 items, which characterize the different parts of each study. One of the items is not scoreable in the field of sports science (item 1). Scores were independently calculated, avoiding any potential bias of the authors. When a study was not available on the PEDro databases, two authors alone (M.J. and A.M.) rated the risk of bias. Disagreements between authors were solved by consensus in a meeting with a third author (R.O.). When this was not achieved, a third investigator (R.O.) was used to carry out the analysis and debate with the first two investigators to reach an agreement.

### 2.5. Certainty Assessment

Based on the Physiotherapy Evidence Database scale and to assess the interventions’ evidence, the [39] criteria were applied. Therefore, the selected studies were grouped by levels of evidence, according to their methodological quality. A study with a PEDro score of 6 or more is considered level 1 (high methodological quality) (6–8: good, 9–10: excellent), and a score of 5 or less is considered level 2 (low methodological quality) (4–5: moderate; <4: poor). Due to the clinical and statistical heterogeneity of the results, a qualitative review was performed, conducting a best-evidence synthesis [40,41]. This classification indicates that if the number of studies displaying the same level of evidence for the same outcome measure or equivalent is lower than 50% of the total number of studies found, no evidence can be concluded regarding any of the methods involved in the study.

## 3. Results

### 3.1. Selection of Studies

**Figure 1:**
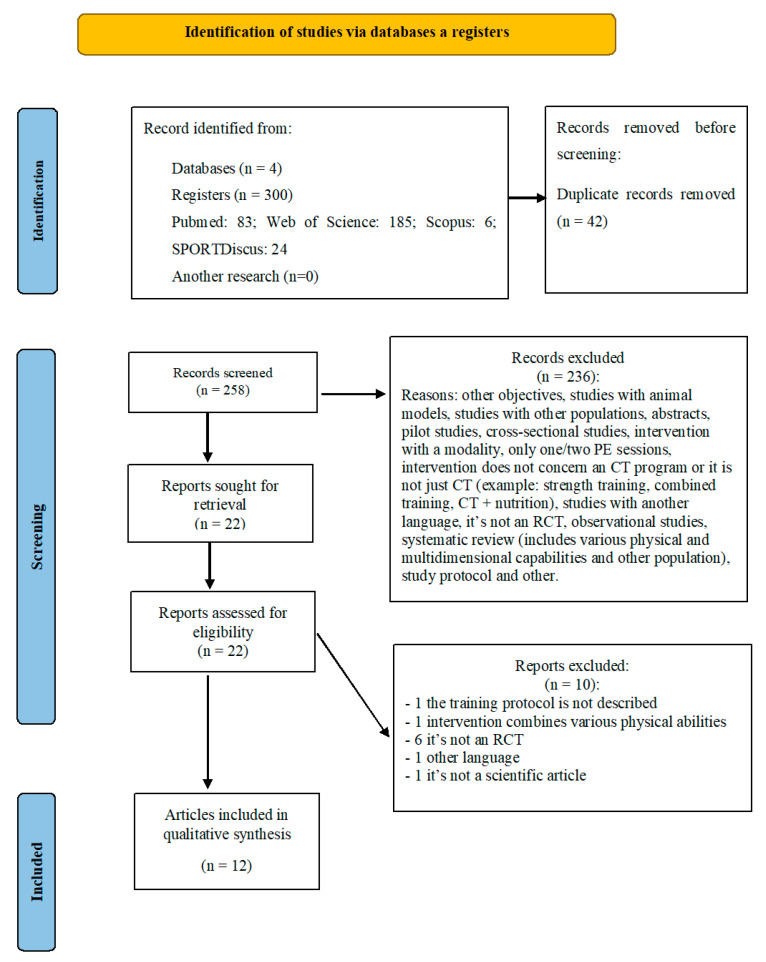
Flow chart of study design by PRISMA 2020.

A total of 258 studies were identified, through research carried out in the databases. In a first phase, which included the reading of titles and abstracts, 22 studies potentially relevant to the study were identified. Considering the previously defined eligibility criteria and after the full reading of the articles, 12 studies were identified as meeting the criteria for inclusion and were assessed for quality using the PEDro scale and included in this systematic review for full analysis. Figure 1 represents the flowchart of this systematic review.

### 3.2. Methodology Quality

The analysis of the quality of the 12 studies presents scores ranging between 4 and 7 on the PEDro scale, showing a moderate to good quality of the methodological procedures [37], as mentioned in Table 2. Items 5 and 6 were not applicable to the studies included.

### 3.3. Studies Characteristics

The Table 3 presents the characteristics of the studies, namely: authors’ names, year of publication, aims, participants, assessment instruments, duration/frequency, exercises, and intensities.

### 3.4. Characteristics of Interventions

Two of the selected studies used both methodologies with interval CT (ICT) and continuous CT (CCT) [42,43], one used a methodology with ICT only [45] and the other nine studies included a CCT [44,46,47,48,49,50,51,52,53].

CCT is a form of exercise performed “continuously without any periods of rest involved”. CCT usually involves cardiorespiratory activities (e.g., walking, running, cycling), in turn, ICT alternates shorts bursts of moderate to intense activity with longer intervals (about 1–2 min) of less intense activity. For example, if the exercise is walking, alternate with short runs [54,55,56].

#### 3.4.1. Participants

From all selected studies, the total number of participants involved was 402, with 186 being included in the intervention groups and 192 in the control groups. The participants included children, adolescents, and adults. Specifically, there were two studies with child participants [44,45]. From the 12 studies selected, nine were carried out in the population with Down syndrome [42,44,45,46,47,50,51,52,53], while only three were carried out with participants with ID [43,48,49].

#### 3.4.2. CT Programs

All selected studies presented assessment protocols, namely the anthropometric assessment of weight and height, which were present in six studies [42,43,45,46,47,51] or the assessment of body composition, such as calculating the BMI, measuring the perimeter of the waist, abdominal, and thigh, presented in six studies [42,43,46,50,51,53].

Considering cardiorespiratory function, six studies used continuous load increment tests [42,43,48,50,51,53] in which two of them also applied the functional test known as the 6-min walk test [42,43].

Only two studies [42,43] assessed neuromuscular capacity using a manual dynamometer. One study also applied the functional test of a 30 s chair stand [42].

Six studies evaluated physiological parameters such as lipid profile, hematological, immunological, and metabolic rate parameters [42,43,48,50,51,53]. Three studies performed the assessment of cognitive ability [46,47,52]. One study assesses manual dexterity and expectation, and another one assessed attention function [49].

#### 3.4.3. Structure (Duration/Frequency)

Regarding the intervention durations, the studies presented programs with a duration of 8 to 15 weeks, with most studies prescribing a duration of 8 to 12 weeks (8, 10, or 12 weeks) [42,44,45,46,47,48,49,50,51,52,53].

Concerning weekly frequency, the studies ranged from 2 to 5 sessions per week, with 3 as the most common weekly frequency [42,44,45,46,47,49,50,51,52,53], lasting from 20 to 60 min, using cycle ergometers (treadmill, exercise bike, elliptical, step, or simply walking).

#### 3.4.4. Continuous Cardiorespiratory Training and Main Results

The CCT method was the most used in the prescription of CT for individuals with ID, being transversal to most of the studies analyzed in this systematic review [42,43,44,46,47,48,49,50,51,52,53].

CCT was carried out through walking, running, or using ergometers such as treadmills, steps, or stationary bikes, in which intensities vary from 70% to 80% VO_2 peak_, 100% to 110% of the ventilatory threshold or between 50% and 75% HR_max_. All studies started the CCT program with low (very light to light) intensity, with the exception of the study by Boer and Moss [42] which started with moderate intensity. All studies prescribed a progressive increase in intensity, (low to moderate and/or moderate to vigorous), with the exception of the studies by Holzapfel [46,47] and Chen [44] where there is no intensity progression.

Regarding the body composition effects of CCT, there was a reduction in body weight, percentage of fat mass, waist circumference, and waist-to-hip-ratio, as shown in Table 4 and Appendix A.

At the same time, as shown in Table 5 and Appendix A, CCT promoted positive changes in the lipid profile, hemodynamic parameters and metabolic markers.

Regarding changes in cardiorespiratory function, as shown in Table 6 and Appendix A, studies reported improvements in several variables.

In the functional capacity assessment tests, performance improvements were reported in the 6 min walk test [42,43], time up and go (8-foot and go) test [42], sit-to-stand [42], and muscle fatigue resistance [43], in accordance with Table 7 and Appendix A.

In tests that assessed cognitive/neural function, the outcomes of measures of visual-perceptual organization, nonverbal reasoning, and trial-and-error learning, improvements were also reported, namely in manual dexterity [44,47], assembly subtest [47], cognitive planning ability [47], semantic language fluency [52], set shifting ability [52] and working memory [46], attention concentration and attention span [49],decrease in reactions times [52], and inhibitory control [52] (see Appendix A).

### 3.5. Interval Cardiorespiratory Training and Main Results

Considering the ICT method, several variables showed improvements which were presented in Table 8 and in Appendix A.

The study by El Kafy and Helal [45] was performed with children participants (8 to 12 years), showing that this age group can also improve their cardiorespiratory function, namely: vital capacity, forced vital capacity, forced expiratory volume, and peak expiratory flow rate.

The studies selected in this review had the following characteristics in ICT: a shorter volume with a duration of 10 s at maximum speed, followed by 90 s of rest [42] or 15 s of full speed with 45 s of rest [43] or 2 min of work for 1 min of rest [45]. In the Boer et al. [43] study, intensity was not quantified, while Boer and Moss [42] used an intensity of 100% of the ventilatory threshold (aerobic threshold), increased up to 110% (anaerobic threshold), and El Kafy and Helal [45] carried out a prescription based on the VO_2 peak_, specifically between 70% to 80% VO_2 peak_.

Regarding the exercise modes, ICT was performed through walking [42], jogging/sprint [43] or using cycle ergometers such as stationary bikes [42] or rowing [45] at 50% to 80% HR_max_ [42,44,45,46,47,50,51,52,53].

The most common parameters used for the prescription and control of the effort intensity were the % HR_max_, with percentages ranging from 50% to 80%; however, a prescription based on VO_2max_ (70% to 80%) or ventilatory threshold (100% to 110%) were also applied [42,43,45].

## 4. Discussion

The present study aimed to describe the effects on the health-related and functional capacity outcomes of CT for individuals with ID as well as to characterize CT programs implemented in these individuals and the type of exercise and the guidelines for the prescription of effective CT programs.

### 4.1. Main Results

The results of this studies showed that CT programs applied in some studies have positive effects on improving cardiorespiratory function [42,43,45,50,53], lipid profile, hemodynamic parameters, metabolic markers [43,48,50,51,53], functional function [42,43], body composition [42,43,48,50,51], and cognitive capacity in people with ID, including Down syndrome, meeting the benefits found for the general population [57,58,59].

From the 12 studies analyzed, two involved children as participants and demonstrated that they, too, could improve cardiorespiratory function [45]. In addition to promoting cardiorespiratory function, the literature shows that CT in children also improves lipid profile, hemodynamic parameters, and metabolic markers [57].

CT also promotes improvements in neuromuscular capacity of upper and lower limbs [42,43]. Neuromuscular capacity was assessed using a manual dynamometer or functional tests such as sit-to-stand [42,43]. Boer and Moss [42] and Boer et al. [43] reported an increase in neuromuscular capacity, especially in the CCT group. Our results are supported in the literature namely by the Konopka and Harber [60] study where authors reported that CT alters protein metabolism and induces skeletal muscle hypertrophy. These findings are important when prescribing a physical exercise program, to achieve maximum benefits.

CT seems to be the best training method to improve some variables such as those indicated in Table 4, Table 5, Table 6, Table 7 and Table 8 and Appendix A [61]. Since these variables were associated with the onset of cardiovascular and metabolic diseases [62,63], their improvement through CT seemed particularly relevant. In recent decades, there has been a significant increase in the years of life expectancy of individuals with ID and with Down syndrome, justifying the greater need to study the effects of intervention strategies that improve health and reduce the impact of comorbidities associated with ageing [64]. According to the results of several studies, the CT seems to be an effective type of exercise in people with ID.

### 4.2. Exercise Prescription

Considering the characterization of CT programs of all studies included in the present systematic review, there were aspects that are common and more evident, such as: (a) duration of 12 weeks; (b) three sessions per week; (c) duration of 20 to 60 min per session, always encompassing the warm-up and cool down phases; (d) exercises performed using ergometers such as cycling exercise, elliptical, stepper, or treadmill walking; (e) intensity between 50% and 80% of HR_max_ or 70% to 80% VO_2max_.

The ACSM [54] most recent recommendations for CT prescription for the population with ID and Down syndrome show an intensity of 40% to 80% of VO_2max_ or HR_max_, 30 to 60 min per session, suggesting activities like walking-based activities and swimming ergometry (arm and leg). The characteristics of the applied intervention programs included in this systematic review were in accordance with international recommendations, namely the ACSM [54] and the National Strength and Conditioning Association—NSCA guidelines [65]. All studies have prescribed intensities recommended by the ACSM [54]; however, all studies started the CT programs at intensity values higher than those recommended by these guidelines. Considering the heterogeneity of the population with ID, the prescription of training intensity may be related to the physiological characteristics of the individuals, namely if they also have Down syndrome or depending on the degree of disability (mild, moderate, severe, and/or profound).

The duration of the CT program interventions analyzed varied between 8 and 12 weeks [42,44,45,46,47,48,49,50,51,52,53]. However, Rodrigues et al. [66], by analyzing the effect of past behavior on future behavior, considering the motivational sequence proposed by self-determination theory, points out that, in terms of adherence and maintenance of a physical exercise behavior, the first 6 months of participation are crucial for the success of the following 6 months. This systematic review reveals a lack of studies with longer duration (six or more months), which is an aspect to be considered in future intervention studies.

### 4.3. Continuous Cardiorespiratory Training vs. Interval Cardiorespiratory Training

The literature showed that two CT methods such as CCT and ICT, can be prescribed for individuals with ID and considered as safe and effective options, given the absence of adverse events, the low dropout rate and excellent adherence to training. Although several studies have implement CCT [42,43,46,47,50,51,52,53], the ICT may be more effective, particularly for individuals with some training experience, since this is a method with higher intensity [42,43]. Nonetheless, CCT is the most recommended by ACSM [54] for the population with ID and Down syndrome. Three days or more of moderate to vigorous CT, 40–80% VO_2 peak_, for 30 to 60 min, using walking, ergometry, or other activities is recommended.

Results of the present systematic review were supported by other studies which showed that exercise intensity is an important factor for the improvement of cardiorespiratory function and reversing the risk factors of the metabolic syndrome [67,68]. A recent meta-analysis [69], compared the use of ICT and CCT methods. ICT results showed a greater increase in peak oxygen uptake, peak heart rate, first ventilatory threshold, and a reduced ejection fraction compared with CCT, in patients with coronary artery disease or heart failure. Another meta-analysis reported a more significant increase in VO_2max_ in ICT when compared with CCT, in healthy, young to middle-aged adults [70]. However, it appears to be more exhausting and stressful for individuals with ID which appear to have a lower degree of resilience to the stress imposed by physical exertion [42,43]. Although the present systematic review was not able to find which type of CT is better, ICT due to the higher exertion intensities would provide better results which should be analyzed in future studies with ID and/or Down syndrome participants.

### 4.4. Physiological Process

During cardiorespiratory training, several physiological processes occur, leading to various physiological adaptations in the human body. With exercise, the human body needs more oxygen in the muscles. To respond to this demand, there is an increase in heart rate, ensuring that the heart pumps more blood. With this physiological response, we expect an increase in cardiac output: the amount of blood pumped per heartbeat. To allow greater blood flow to the muscles, the blood vessels dilate, allowing for increased blood flow (oxygen and nutrients needed for activity). In turn, for greater oxygen uptake, lung ventilation increases. During exercise, energy expenditure increases, also increasing heat production. To keep the body at a healthy temperature, the body responds by sweating. Regular cardiorespiratory training, by triggering this whole process, affects cardiorespiratory capacity and can lead to the other adaptations mentioned above [54].

### 4.5. Limitations and Future Research

The authors of the studies included in this systematic review highlight some limitations found in their studies, which should be taken into account for future studies, such as lack of information or analysis regarding the level of DI [42]; difficulties in randomization [43,46,52]; associated comorbidities (e.g., cardiovascular diseases) [43]; lack of validity and reliability of some applied tests [43]; lack of quantification of physical activity practice [44]; small sample size [44,49]; large age range [49]; short duration of the intervention program [51]; lack of analysis of intervention detraining [51,52]; and measures of assessment susceptible to media errors [53].

This systematic review shows a lack of studies on the effects of applying a CT program in individuals with ID but without another associated conditions. At the same time, no intervention studies were found for the elderly population with ID which leads us to suggest the application of CT programs in older people with ID. Although the ACSM [54] does not distinguish between age groups, it will be interesting in the future to realize that the guidelines and benefits are transversal for all. Equally, more randomized studies are needed to assess the variables shown in Table 4, Table 5, Table 6, Table 7 and Table 8 and in non-randomized controlled trials studies since their programs are quite different which difficult comparisons between studies. The longer implementation of CT programs will also allow the knowledge enlightenment regarding training methods, programs structure, type of exercise, and periodization in order to have more adapted and effective exercise prescriptions, as some of the barriers to physical activity are also reduced [26]. Although we know that individuals with Down syndrome have different physiological responses than individuals with just ID due to an underlying autonomic dysfunction [71] and taking into account the heterogeneity of assessment methodologies and variables evaluated, we cannot conclude whether the results are different in such individuals. However, the ACSM [54] does not make this differentiation either. It is important to take special care in the prescription and implementation of CT programs in the population with Down syndrome, due to its atlantoaxial instability [72]. To increase physical fitness, namely cardiorespiratory fitness or VO_2max_, Swain and Franklin [73] suggested that the intensity of the exercise should vary according to the participant initial fitness level.

The present systematic review showed some limitations similar to those recently reported by Jacinto et al. [74] concerning strength training for individuals with ID, such as (i) heterogeneity of different studies; (ii) little clearness in some studies regarding the randomization process; (iii) absence of follow-up; (iv) different assessment methodologies which did not allow further discussion or a meta-analysis about the CT effects produced; and (v) the level of ID was not mentioned in all studies included, which limits the generalization of the results and recommends such description in future studies.

### 4.6. Practical Implications

The present study includes aspects and recommendations that PE professionals should consider when structuring, prescribing, and implementing a CT program. For a population where a sedentary lifestyle prevails, with several associated comorbidities, the characteristics presented in this document become essential to promote the expected benefits and results, namely the maintenance/increase of physical fitness, quality of life, and health, thus decreasing, the risk of onset of chronic diseases. Along with the ACSM recommendations, to which this document is intended to be a supplement, the conditions for successful evaluation, prescription, implementation, and follow-up of CT in individuals with ID are met.

Associated with an appropriate lifestyle, the implementation of CT programs, incorporated into the routine of the target population, provokes a set of adaptations and benefits, promoting healthy aging and fuller health.

## 5. Conclusions

This manuscript includes a review of studies published in recent years about an in-depth analysis of the basic guidelines for prescribing CT in individuals with ID and Down syndrome, and about its main benefits for health and well-being. In summary, the basic aspects for CT prescription are:

(i). Duration of 8 to 12 weeks (we emphasize that the essential is that the CT programs are implemented continuously);
(ii). Frequency of three sessions per week;
(iii). Duration of 20 to 60 min per session, always taking into account the warm-up and cool down phases;
(iv). Exercises performed using ergometers such as cycling exercise, elliptical, stepper, or treadmill walking;
(v). Intensity between 50% to 80% of HR_max_ or 70% to 80% VO_2max_.

Although CCT is more frequently prescribed and recommended, ICT also seems to be a good option and more effective in variables such as peak oxygen uptake, peak heart rate, first ventilatory threshold, and a reduced ejection fraction. However, it should only be prescribed with some previous training experience, as it is a more exhausting and intense training method.

This systematic review also shows that CT promotes benefits in cardiorespiratory function, lipid profile, and hemodynamic and metabolic markers which have direct effects on body composition, as well as an increase in upper and lower limb strength and cognitive/neural capacity in individuals with ID and inclusive Down syndrome.

## Figures and Tables

**Table 1:** Research strategy.

Search Number	Research Content
1	(aerobic exercise* OR aerobic training* OR cardio training* OR cardiorrespiratory training* OR cardiorespiratory training* OR cardio exercise* OR cardiorrespiratory exercise* OR cardiorespiratory exercise* OR continuous exercise* OR continuous training* OR high-intensity interval training* OR HIIT* OR interval training* OR interval exercise*) AND (“mental retardation” OR “intellectual disability” OR “intellectual disabilities” OR “intellectual and developmental disabilities” OR “Down syndrome”)

**Table 2:** Assessment of the quality of articles through the PEDro scale.

Author (Year)	PEDro Scale											Total Score	Methodological Quality
	1	2	3	4	5	6	7	8	9	10	11		
Boer and Moss [42]	S	1	0	1	0	0	1	1	1	1	1	7	Good
Boer et al. [43]	S	1	0	1	0	0	0	1	0	1	1	5	Moderate
Chen et al. [44]	S	1	0	1	0	0	0	1	1	1	1	6	Good
El Kafy and Helal [45]	S	1	0	1	0	0	0	0	0	1	1	4	Moderate
Holzapfel et al. [46]	S	0	0	1	0	0	0	1	1	1	1	5	Moderate
Holzapfel et al. [47]	S	0	0	1	0	0	0	1	1	1	1	5	Moderate
Kim et al. [48]	S	1	0	1	0	0	0	0	0	1	1	4	Moderate
Li et al. [49]	S	1	0	1	0	0	0	1	1	1	1	6	Good
Ordoñez et al. [50]	N	1	0	1	0	0	0	0	0	1	1	4	Moderate
Ordonez et al. [51]	S	1	0	1	0	0	0	0	0	1	1	5	Moderate
Ringenbach et al. [52]	N	1	0	1	0	0	0	0	0	1	1	4	Moderate
Rosety-Rodriguez et al. [53]	N	1	0	1	0	0	0	1	0	0	1	4	Moderate

Notes: 1, yes; 0, no (item 1, not scoreable).

**Table 3:** Characteristics of cardiorespiratory training programs.

Studies	Aims	Participants	Duration/Frequency	Exercise/Intensity	Measurements
Boer and Moss [42]	Effects of training on health anthropometrical, physical and functional parameters in participants from South Africa.	N = 42 (♂ = 25; ♀ = 17); 33.8 ± 8.6 y; 18–50 y; Down syndrome; Randomized groups: ICT (N = 13), CCT (N = 13) and CG (N = 16).	12 weeks; 3 × week; 30 min/session.	CCT: Walking or cycling 70–80% VO_2 peak_; ICT: 10–30 s all out sprints with 90 s (1:3 work-rest ratio) of low cadence, low intensity walking or cycling.	Body weight—electronic scale (Beurer, Germany); Height—stadiometer (Siber-Hegner GPM, Switzerland); BMI (kg/m^2^); Waist circumference—tape (Lufkin, Cooper Tools, Apex, NC, USA); Hip circumference—tape; Fat mass and fat-free mass– bioelectrical impedance (Bodystat 1500 MDD, Douglas, Isle of Man, UK); Systolic and diastolic Blood pressure–sphygmomanometer (MicroLife, Widnau, Switzerland); Blood samples (finger prick); Total cholesterol, glucose (Roche Diagnostic Mannheum, Germany); Hand grip strength (hand dynamometer—Takei, Grip D, T.K.K.5401; Niigata City, Japan); 6-min walk distance, Sit-to-stand, Up and go test; VO_2 peak_ (MetaLyzer 3B system—Cortex, Leipzig, Germany); Electrocardiogram (Custo Med, Schiller, Switzerland).
Boer et al. [43]	Effect of sprint interval training on metabolic and physical fitness in participants from South Africa.	N = 46; 17 ± 3 y; ID; Randomized groups: ICT (N = 17); CCT (N = 15) e CG (N = 14)	15 weeks; 2 × week; 40 min/session.	ICT: sprint interval block (10 min) CCT (10 min), another sprint interval block (10 min); Each sprint interval block consisted of 10 sprint bouts (>100 r/min) of 15 s at a resistance matching with the ventilatory threshold (VTR), alternated with 45 second’s relative rest (50 r/min at VTR); Starting from week 8 the intensity was increased up to 110% of VTR; CCT: 3 blocks of 10 min (cycling, running, stepping); ventilatory threshold (60 r/min), which was increased to 110% of ventilatory threshold from week 8 onwards.	Stadiometer; Digital balance scale; BMI; Waist circumference; BIA; VO_2 peak_; Electrocardiogram; Sphygmomanometer; Respiratory gas (Metalyzer 3B); 6-min walk test, Sit-to-stand test; Hand grip strength (hand dynamometer); Lipid profile (Roche Diagnostic kits).
Chen et al. [44]	Effectiveness of assisted therapy (ACT) on manual dexterity in older adults with Down syndrome	N = 17; 32 to 52 y; Down syndrome; Randomly assigned to assisted cycling therapy (N = 9, 39.74 ± 10.08 y) or voluntary cycling (N = 8, 40.5 ± 8.55 y).	8 weeks; 3 × week; 30 min/session.	Specialized stationary recumbent cycle ergometer (Theracycle, Exercycle Company, Franklin, MA, USA); Voluntary cycling: participants pedaled at a self-selected rate Assisted cycling therapy: 35% faster than the voluntary cadence;	Purdue Pegboard Test (Model # 32020A, Lafayette Instruments Company, Lafayette, IN, USA); Exercise Perception.
El Kafy and Helal [45]	Effects of a rowing exercise regimen vs. a chest physical therapy program on pulmonary function in Egyptian children.	N = 29; 9.36 ± 1.35 y; 8–12 y; Down syndrome; Randomized groups: Chest physical therapy (N = 15, 9.22 ± 1.3 y) and rowing ergometer (N = 14, 9.5 ± 1.4).	12 weeks; 3 × week; 20 to 30 min/session.	Rowing ergometer; Level of resistance: 1 to 4; 2-min work and 1-min rest; 50% to 80% HRmáx.	Weight and height scale; Vital capacity, forced vital capacity, forced expiratory volume after 1 s, and peak expiratory flow rate (PEFR)—Zan-680 Ergospirometry; Rowing ergometer (Kettler Coach, 8 speeds, Henze Kettler GMPH and Co. D 59469. ENSE-PARSIT—Type 7985, Germany).
Holzapfel et al. [46]	Effects assisted cycling therapy on short-term and working memory in USA adolescents.	N = 44; Down syndrome; Randomly assigned to assisted cycling therapy (N = 17, 19.4 ± 4.9 y) or voluntary cycling (N = 16, 18.4 ± 3.4 y). Non-randomized CG (N = 11, 17.2 ± 4.3).	8 weeks; 3 × week; 35 min/session.	Modified motorized stationary recumbent bicycle (Exercycle; Franklin, MA, USA); Voluntary cycling: participants pedaled at a self-selected rate Assisted cycling therapy: 35% to 180% faster than the voluntary cadence; not exceed 60% HRmáx or 95 rpm.	Assess verbal mental age—The Peabody Picture Vocabulary Test IV (PPVT-IV; Dunn and Dunn, 2007); Height and weight were measured to obtain the BMI (kg/m^2^); Short-term and WM assessment—verbal memory digit span memory test (Lezak, 2004; Wechsler Memory Scale III, The Psychological Association, 1997).
Holzapfel et al. [47]	Effects of eight weeks of voluntary cycling, and no cycling on manual dexterity and cognitive planning ability in USA adolescents.	N = 48; Down syndrome; Randomly assigned to assisted cycling therapy (N = 18, 19.4 ± 4.9 y) and voluntary cycling (N = 16; 18.4 ± 3.4 y). Non-randomized CG (N = 14, 17 ± 4 y).	8 weeks; 3 × week; 30 min/session.	Modified motorized stationary recumbent bicycle (Exercycle; Franklin, MA, USA); Voluntary cycling: participants pedalled at a self-selected rate Assisted cycling therapy: 35% to 80% more faster than the voluntary cadence; not exceed 60% HRmáx or 95 rpm.	Weight; Height; Manual dexterity—PPT (Lafayette Instrument Company); Executive function test of cognitive planning ability- ToL (Jurado and Rosselli, 2007; Lezak, 1995); Handedness, vision, hearing, and verbal mental age—(Oldfield, 1971), a standard or modified Snellen Eye Chart, an audiometer (Maico Ma 25), and the Peabody Picture Vocabulary Test 4th ed. (PPVT-IV; Dunn and Dunn, 2007);
Kim et al. [48]	Effects of training and half-bath on body composition, cardiorespiratory system, and arterial pulse wave velocity in participants from Republic of Korea.	N = 24; ID; Randomized groups: CCT (N = 8, 19.3 ± 1.2 y), half-bath group (N = 8, 18.9 ± 2.5 y) and CG (N = 8, 20.2 ± 1.1 y).	12 weeks; 5 × week; 50 min/session.	Two 15-min exercise periods; treadmill and stationary bicycle; 50–70% HRmáx	BIA; Balke-Ware protocol; Heart rate, respiratory, and circulatory variables (electrocardiograph instrument); Gas analyzer (Quark b2, Italy); Vessel compliance (vessel compliance equipment—PWV 3.0-K_M TEC, Republic of Korea).
Li et al. [49]	Impact of moderate-intensity aerobic exercise combined with acupuncture on the attention function	N = 48: ID; Randomized groups: Traditional Chinese Medicine acupuncture group (N = 12, 14.7 ± 1.6 y), CCT (N = 12, 13.9 ± 1.2 y), exercise and acupuncture combined intervention group (N = 12, 14.7 ± 1.6 y) and CG (N = 12, 14.4 ± 1.2 y).	12 weeks; 3 × week; 50 min/session.	Treadmill—moderate sustained exercise at the target intensity of 65% to 75% HRmáx	An eye tracker (Tobii Pro spectrum, Tobii Pro, Stockholm, Sweden) and test software (Tobii Pro lab, version 1.162, Stockholm, Sweden) were used for the attention function test.
Ordoñez et al. [50]	Influence of aerobic training on plasma adipokines in Spain obese women.	N = 20 ♀; 18–30 y; Down syndrome; Randomized group: CCT (N = 11, 24.7 ± 3.6 y) and CG (N = 9, 25.1 ± 3.9).	10 weeks; 3 × week; 60 min/session.	Ergometer session (treadmill); 55–65% HRmáx.	Maximal continuous incremental test on a treadmill; Fat-mass percentage—bioelectrical impedance analysis (Tanita TBF521); Waist and hip circumference—anthropometric tape (Holtain Ltd.); Blood samples were collected from the antecubital vein; Plasma levels of adiponectin and leptin—immunoenzymatic methods using commercial ELISA kits (R&D, Minneapolis, MN, USA).
Ordonez et al. [51]	Influence of aerobic training on pro-inflammatory cytokines and acute phase proteins.	N = 20 ♀; 18–30 y; Down syndrome; Randomized group: CCT (N = 11, 24.7 ± 3.6 y) and CG (N = 9, 25.1 ± 3.9).	10 weeks; 3 × week; 30 a 40 min/session.	Treadmill; 55% to 65% HRmáx.	Continuous maximal incremental test on a treadmill; Gas exchange—breath metabolic system; Electrocardiogram—12 lead stress analysis system; Fat mass percentage—bioelectrical impedance (Tanita TBF521); Fat-free—prediction equations BMI [=weight (kg)/height (m)^2^]; Height—stadiometer; Body weight—electronic balance; Waist circumference and hip circumference—anthropometric tape (Holtain Ltd.); Blood samples—antecubital vein; Plasmatic levels of tumour necrosis factor (TNF)-α, interleukin (IL)-6, a1-antitrypsin and fibrinogen were assessed by commercial enzyme-linked immunosorbent assay kits (Immunotech, Westbrook, MA, USA); C-reactive protein—nephelometry on a BN-II analyser (Dade-Behring Diagnostics, Marburg, Germany).
Ringenbach et al. [52]	Effects of 8 weeks of assisted cycling therapy on measures of reaction times, set-shifting, inhibition and language fluency.	N = 44; ♂ = 25; ♀ = 19; Down syndrome; Randomized group: assisted cycling therapy (N = 17, 19.4 ± 4.9 y) and voluntary cycling (N = 16, 18.4 ± 3.4 y). Non-randomized CG (N = 11, 17.2 ± 4.3).	8 weeks; 3 × week; 30 min/session.	Modified Theracycle (Exercycle, Franklin, MA, USA); Assisted cycling therapy: 35% faster than the voluntary cadence during the 5-min warm-up. From session to session, but not within sessions, the cadence was increased by 3 to 5 rpm; Voluntary cycling: o pedal at a self-selected rate.	Information processing speed was assessed through simple reaction times—reaction time to visual stimulus (Lafayette, IN, EUA); Set-shifting ability—modified Wisconsin Card Sorting Test (MCST) adapted from Wilson et al. (1996); Response inhibition was assessed with the NEPSY Knock-Tap task (KT; Korkman et al. 1998); Language fluency (NEPSY, Pennington et al. 2003).
Rosety-Rodriguez et al. [53]	Determine how long the anti-inflammatory effect induced by CT is maintained in Spain participants.	N = 20 ♀; 18 to 30 y; Down zyndrome; Randomized group: CCT (N = 11, 24.7 ± 3.6 y) and CG (N = 9, 25.1 ± 3.9 y).	10 weeks; 3 × week; 60 min/session.	Treadmill: 30–40 min (increasing 2-min and half each two weeks) at a work intensity of 55–65% of peak heart rate (increasing a 2.5% each two weeks).	Fat mass percentage and visceral fat—bioelectrical impedance (Tanita, IL, USA) TBF521); Plasma level of IL-6 was assessed by commercial ELISA kits (Immunotech, MA, USA); High-sensitive C-reactive protein (hs-CRP) in plasma was assessed by nephelometric methods on a BN-II analyser (Dade-Behring Diagnostics, Marburg, Germany); Maximal continuous incremental test on a treadmill; Waist and hip circumference.

BIA—bioelectric impedance; BMI—body mass index; CG—control group; CT—cardiorespiratory training; CCT—continuous cardiorespiratory training; ICT—interval continuous training; min—minutes; N—participants; s—seconds; VO_2 peak_—peak oxygen consumption; HR_máx_—maximum heart rate; rpm—rotations per minute; y—years; ♂—male; ♀—female.

**Table 4:** Percentage change in anthropometric variables with CCT programs intervention.

Author	Variable	Percentage
Boer and Moss [42]	Weight (kg)	−1.4
Kim et al. [48]		−7
Boer et al. [43]	Fat mass (%)	−3.2
Kim et al. [48]		−17.4
Ordonez et al. [51]		−11.1
Ordoñez et al. [50]		−11.1
Boer et al. [43]	Waist circumference (cm)	−2.7
Ordonez et al. [51]		−3.5
Ordoñez et al. [50]		−3.5
Ordonez et al. [51]	Waist-to-hip-ratio	−12
Ordoñez et al. [50]		−12

**Table 5:** Percentage change in lipid profile, hemodynamic parameters and metabolic markers with CCT programs.

Author	Variable	Percentage
Ordoñez et al. [50]	Plasma leptin levels (ng/mL)	−18.6
Ordonez et al. [51]	Plasmatic levels of tumour necrosis factor (pg/mL)	−27.1
Ordonez et al. [51]	High sensitive C-reactive protein (mg/dL)	−17
Rosety-Rodriguez et al. [53]		−17
Ordonez et al. [51]	Interleukin−6 (pg/mL)	−34.4
Rosety-Rodriguez et al. [53]		−34.4
Boer et al. [43]	Homeostasis model assessment of insulin resistance	−11.5
Ordonez et al. [51]	Fibrinogen (g/L)	−15.2
Kim et al. [48]	Pulse wave velocity (m/s/height)	−6.5

**Table 6:** Percentage gained of cardiorespiratory outcomes with CCT programs.

Author	Variable	Percentage
Boer and Moss [42]	VO_2 peak_ (L/min)	6.4%
Kim et al. [48]		24%
Rosety-Rodriguez et al. [53]		14.8%
Ordoñez et al. [50]	Maximum oxygen uptake (mL/kg/min)	14.8%
Boer and Moss [42]	Rel. peak VO_2_ (mL/kg/min)	4.8%
Kim et al. [48]	HR_max_	3.9%
Boer et al. [43]	Ventilatory threshold (w)	8.1%
Boer et al. [43]	Ventilatory threshold (VO_2_, L/min)	6.6%
Boer and Moss [42]	Ventilatory threshold (VO_2_, L/min)	16.2%
Boer and Moss [42]	Time to exhaustion (s)	13.8%

**Table 7:** Percentage changes in the functional capacity tests performance with CCT programs intervention.

Author	Variable	Percentage
Boer and Moss [42]	6-min walk test (m)	11.4%
Boer et al. [43]		12.9%
Boer and Moss [42]	8-ft up and go (s)	−22.9%
Boer and Moss [42]	Sit-to-stand (amount/30 s)	13.8%
Boer et al. [43]	Muscle fatigue resistance (s)	13.3%

**Table 8:** Health related outcomes of ICT programs.

Author	Variables	Variable	Percentage
Boer and Moss [42]	Anthropometry	Weight (kg)	−3.3%
Boer and Moss [42]		BMI (kg/m^−2^)	−2.8%
Boer et al. [43]		Waist circumference (cm)	−4.7%
Boer et al. [43]		Fat (%)	−12.5%
Boer et al. [43]	Lipid profile, hemodynamic parameters and metabolic markers	Cholesterol (mg/dL)	−11.6%
Boer et al. [43]		HDL (mg/dL)	7.6%
Boer et al. [43]		LDL (mg/dL)	−10%
Boer et al. [43]		Triglyceriden (mg/dL)	−11.9%
Boer et al. [43]		Fasting nsulin (IU/mg)	−27.3%
Boer et al. [43]		Insulin resistance (HOMA-IR))	−26.1%
Boer et al. [43]	Cardiorespiratory function	Systolic blood pressure (mmHg)	−9.7%
Boer and Moss [42]		VO_2 peak_ (L/min)	15.4%
Boer et al. [43]			7.7%
Boer et al. [43]		Peak power (w)	13.3%
Boer and Moss [42]		Time to exhaustion (s)	18.8%
Boer and Moss [42]		Rel. peak VO_2_ (mL/kg/min)	12.1%
Boer and Moss [42]		Ventilatory threshold (L/min)	23.6%
Boer et al. [43]		Ventilatory threshold (w)	13.3%
Boer et al. [43]		Ventilatory threshold (w and L/min)	11.1%
El Kafy and Helal [45]		Vital capacity (L)	6.4%
El Kafy and Helal [45]		Forced vital capacity (L)	7.4%
El Kafy and Helal [45]		Forced expiratory volume (L)	7.2%
El Kafy and Helal [45]		Peak expiratory flow rate (L/min)	6.2%
Boer and Moss [42]	Functional capacity	6-min walk test (m)	11.4%
Boer et al. [43]			10.2%
Boer and Moss [42]		8-ft up and go (s)	−22.9%
Boer and Moss [42]		Sit-to-stand (amount/30 s)	13.8%
Boer et al. [43]		Muscle fatigue resistance (s)	31.2%

## Data Availability

The data presented in this study are available on request from the corresponding author.

## Appendix A

**Table A1:** Effects of CT programs in people with ID.

Author, Year	Variables on Which CT Had an Impact	Group	Main Results (M ± SD)		*p* Value	*p* Value	*p* Value
			Pre	Post		(vs. CG)	(vs. Other Group)
Boer et al. [42]	**Outcomes of anthropometry**						
	Weight (kg)	CCT	70.2 ± 14.6	69.2 ± 14.6	-	<0.05	-
		ICT	71.7 ± 8.4	69.4 ± 8.3	-	<0.05	<0.05 (vs. CCT)
		CG	74.8 ± 8.4	74.1 ± 8.4	-	-	-
	BMI (kg/m^2^)	CCT	30.6 ± 6.1	30.2 ± 6.3	-	-	-
		ICT	29.3 ± 4	28.5 ± 5	-	<0.05	<0.01 (vs. CCT)
		CG	31.2 ± 4.6	30.9 ± 4.2	-	-	-
	**Outcomes of fitness capacity**						
	8-ft up and go (s)	CCT	5.9 ± 1.2	4.8 ± 0.9	-	<0.05	-
		ICT	5.8 ± 1.9	4.9 ± 1.1	-	-	-
		CG	6.5 ± 1.3	6.2 ± 1.3	-	-	-
	Sit-to-stand (amount/30 s)	CCT	13.1 ± 2	15.2 ± 1.8	-	<0.05	-
		ICT	14.4 ± 2.3	15.5 ± 1.8	-	-	-
		CG	13.1 ± 2.7	13.3 ± 2.3	-	-	-
	6-min walk distance (m)	CCT	499 ± 77.5	563.2 ± 74.9	-	<0.05	-
		ICT	519.7 ± 82.8	562.6 ± 81.7	-	-	-
		CG	476.2 ± 83.5	495.9 ± 85.2	-		-
	**Outcome of cardiorespiratory function**						
	Peak VO_2_ (L/min)	CCT	32.2 ± 7.1	34.4 ± 7.5	-	<0.05	-
		ICT	31.9 ± 7.9	37.3 ± 7.9	-	<0.05	<0.05 (vs. CCT)
		CG	32.1 ± 7.1	30.7 ± 6.1	-	-	-
	Time to exhaustion (s)	CCT	700.5 ± 170.2	813.1 ± 79.4	-	<0.05	-
		ICT	686.8 ± 180.7	845.4 ± 86.3	-	<0.05	-
		CG	706.8 ± 142	699.1 ± 134.8	-	-	-
	Rel. peak VO_2_ (mL/kg/min)	CCT	2200.6 ± 457.5	2312,3 ± 447.3	-	<0.05	-
		ICT	2267.1 ± 578.9	2578.3 ± 508.3	-	<0.05	-
		CG	2363.1 ± 533.1	2271.2 ± 499.9	-	-	-
	Ventilatory threshold (L/min)	CCT	67 ± 15.4	80 ± 13.3	-	<0.05	-
		ICT	67.8 ± 17	88.8 ± 19.4	-	<0.05	-
		CG	69.9 ± 15.9	72.9 ± 15.2	-		-
Boer et al. [43]	**Outcomes of anthropometry**						
	Waist circumference (cm)	CCT	95.9 ± 9.6	93.4 ± 9.6	-	<0.05	-
		ICT	95.8 ± 13.1	91.5 ± 13.1	-	<0.05	-
		CG	95 ± 8.8	95.9 ± 8.2	-	-	-
	Fat (%)	CCT	32.3 ± 7	31.3 ± 6.6	-	<0.05	-
		ICT	34.2 ± 6.9	30.4 ± 7	-	<0.05	<0.05 (vs. CCT)
		CG	32 ± 7.1	32 ± 7	-	-	-
	**Lipid profile, hemodynamic and metabolic markers**						
	Cholesterol (mg/dL)	CCT	162.9 ± 26.6	164 ± 31.3	-	-	-
		ICT	169.8 ± 25.2	154.8 ± 22.9	-	<0.05	-
		CG	169.6 ± 29.7	171.9 ± 25.8	-	-	-
	HDL (mg/dL)	CCT	48.9 ± 9	49.7 ± 10.6	-	-	-
		ICT	54.9 ± 13.5	59.4 ± 11.4	-	<0.05	-
		CG	59.3 ± 16.9	55.9 ± 15.6	-	-	-
	LDL (mg/dL)	CCT	96.4 ± 24.8	97.4 ± 28.5	-	-	-
		ICT	105.2 ± 12.4	95.6 ± 9.3	-	<0.05	<0.05 (vs. CCT)
		CG	92.6 ± 23.5	96 ± 21.2	-	-	-
	Triglycerides (mg/dL)	CCT	91.5 ± 50.4	87.5 ± 50.3	-	-	-
		ICT	79.2 ± 22.2	70.8 ± 16.7	-	<0.05	-
		CG	96.6 ± 75.6	95 ± 85.6	-	-	-
	Fasting insulin (IU/mg)	CCT	13 ± 5.3	12 ± 4.7	-	-	-
		ICT	14 ± 5.9	11 ± 4	-	<0.05	<0.05 (vs. CCT)
		CG	12 ± 3.6	13 ± 3.7	-	-	-
	Homeostasis model assessment of insulin resistance	CCT	2.9 ± 1.3	2.6 ± 1.1	-	<0.05	-
		ICT	2.9 ± 1.3	2.3 ± 0.8	-	<0.05	-
		CG	2.6 ± 0.8	2.7 ± 0.9	-	-	-
	Systolic blood pressure (mmHg)	CCT	121 ± 11	119 ± 9	-	-	-
		ICT	124 ± 10	113 ± 8	-	<0.05	<0.05 (vs. CCT)
		CG	118 ± 10	119 ± 10	-	-	-
	**Outcomes of fitness capacity**						
	Muscle fatigue resistance (s)	CCT	19.5 ± 9.9	22.5 ± 10.9	-	<0.05	-
		ICT	13.7 ± 7.5	19.9 ± 6.8	-	<0.05	-
		CG	21.3 ± 13.8	19.2 ± 11.4	-	-	-
	6-min walk distance (m)	CCT	538.7 ± 105	619 ± 72.2	-	<0.05	-
		ICT	598.2 ± 63.6	665.9 ± 69.4	-	<0.05	-
		CG	567 ± 69.4	591.8 ± 82.7	-	-	-
	**Outcome of cardiorespiratory function**						
	Peak VO_2_ (L/min)	CCT	2.5 ± 0.6	2.4 ± 0.6	-	-	-
		ICT	2.4 ± 0.7	2.6 ± 0.6	-	<0.05	<0.05 (vs. CCT)
		CG	2.3 ± 0.6	2.2 ± 0.5	-	-	-
	Peak power (w)	CCT	179 ± 42.6	178.7 ± 42.7	-	-	-
		ICT	155 ± 36.6	178.8 ± 41.3	-	<0.05	<0.05 (vs. CCT)
		CG	166.8 ± 45.7	158.9 ± 46.8	-	-	-
	Ventilatory threshold (w)	CCT	90.3 ± 24.5	98.3 ± 20.3	-	<0.05	-
		ICT	99.7 ± 25.1	120.6 ± 32.2	-	<0.05	<0.05 (vs. CCT)
		CG	86.1 ± 27.9	82.9 ± 22.7	-	-	-
	Ventilatory threshold (VO_2_)	CCT	1.4 ± 0.3	1.5 ± 0.3	-	<0.05	-
		ICT	1.6 ± 0.5	1.8 ± 0.5	-	<0.05	<0.05 (vs. CCT)
		CG	1.2 ± 0.3	1.2 ± 0.3	-	-	-
Chen et al. [44]	**Outcome of manual dexterity**						
	Dominant Hand Subtest	CCT	-	-	>0.05	-	-
		Assisted Cycling Therapy	-	-	>0.05	-	-
	Non-dominant Hand Subtest	CCT	-	-	<0.05	-	-
		Assisted Cycling Therapy	-	-		-	-
	Bimanual Subtest	CCT	-	-	<0.05	-	-
		Assisted Cycling Therapy	-	-		-	-
	**Outcome of expectation**					-	-
	Expectations	CCT			<0.05	-	-
		Assisted Cycling Therapy				-	-
El Kafy and Helal [45]	**Outcome of cardiorespiratory function**						
	Vital capacity (L)	ICT	1.61 ± 0.02	1.72 ± 0.03	<0.0001	>0.05	-
		CG	1.63 ± 0.03	1.69 ± 0.36	<0.0001	-	-
	Forced vital capacity (L)	ICT	1.38 ± 0.02	1.49 ± 0.03	<0.0001	>0.05	-
		CG	1.39 ± 0.02	1.48 ± 0.02	<0.0001	-	-
	Forced expiratory volume (L)	ICT	1.29 ± 0.03	1.39 ± 0.02	<0.0001	>0.05	-
		CG	1.28 ± 0.03	1.38 ± 0.02	<0.0001	-	-
	Peak expiratory flow rate (L/min)	ICT	171.03 ± 1.13	182.3 ± 2.82	<0.0001	>0.05	-
		CG	170.29 ± 1.37	180.64 ± 2.76	<0.0001	-	-
Holzapfel et al. [46]	**Outcome of cognition function**						
	Working memory	CCT	0.76 ± 1.05	1.65 ± 1.54	<0.003	<0.019	-
		Voluntary CG	1.19 ± 1.11	1.06 ± 1.12	>0.05	-	-
		CG	1 ± 1.34	0.91 ± 1.14	>0.05	-	-
Holzapfel et al. [47]	**Outcome of cognition function**						
	PPT change scores (%) Unimanual right hand	CCT	8.8 ± 18.9	-	-	<0.007	-
		Voluntary CG	9.9 ± 25.6	-	-	<0.005	-
		CG	−1.6 ± 38.7	-	-	-	-
	PPT change scores (%) Unimanual left hand	CCT	6.6 ± 17.2	-	-	<0.006	-
		Voluntary CG	6.2 ± 21.1	-	-	<0.020	-
		CG	−5.3 ± 19.6	-	-	-	-
	Combined unimanual and bimanual (RLB) score	CCT	16.5 ± 23.3	-	-	<0.05	-
		Voluntary CG	11.6 ± 26.2	-	-	<0.034	-
		CG	1.5 ± 68.2	-	-	-	-
	Assembly sub-test	CCT	12.2 ± 14.4	-	-	-	<0.007 (vs. voluntary CT)
		Voluntary CG	5.9 ± 24.4	-	-	-	-
		CG	−2.9 ± 22.8	-	-	-	-
	Cognitive planning ability (measured by the Tower of London test)	CCT	-	-	-	-	<0.05 (vs. voluntary CT)
		Voluntary CG	-	-	-	-	-
		CG	-	-	-	-	-
Kim [48]	**Outcomes of anthropometry**						
	Weight (kg)	CCT	65.6 ± 1.5	61.3 ± 1.6	<0.05	<0.05	-
		Half bath group	66.8 ± 1.9	63.1 ± 1.1	<0.05	-	-
		CG	65.5 ± 1.4	66.1 ± 0.5	-	-	-
	Body fat (%)	CCT	32.3 ± 1.6	27.5 ± 1.1	<0.05	<0.01	<0.01 (vs. half bath group)
		Half bath group	3.3 ± 1.5	29.7 ± 1.6	<0.05	-	-
		CG	32.4 ± 1.1	33.1 ± 1	-	-	-
	**Outcome of cardiorespiratory function**						
	VO_2max_ (mL/kg/min)	CCT	25.12 ± 1.21	33.07 ± 2.37	<0.05	-	-
		Half bath group	26.18 ± 1.2	27.07 ± 1.1	>0.05	-	-
		CG	27.13 ± 1.01	27.05 ± 1	-	-	-
	HR_max_ (beat/min)	CCT	164.51 ± 1.88	171.22 ± 2.77	<0.05	-	-
		Half bath group	165.87 ± 1.05	167.55 ± 1.4	>0.05	-	-
		CG	166.77 ± 1.2	166.81 ± 1.04	-	-	-
	Pulse wave velocity (m/s/height)	CCT	1.63 ± 0.03	1.53 ± 0.06	-	<0.05	<0.05 (vs. half bath group)
		Half bath group	1.62 ± 0.05	1.57 ± 0.03	-	<0.05	-
		CG	1.64 ± 0.04	1.64 ± 0.03	-	-	-
Li et al. [49]	**Outcomes of attention concentration**						
	Attention concentration	Traditional Chinese Medicine acupuncture group	-	-	>0.05	<0.05	-
		CCT	-	-	<0.05	<0.05	-
		exercise and acupuncture combined intervention group	-	-	<0.05		<0.05 (vs. CCT and Traditional Chinese Medicine acupuncture group)
		CG	-	-	>0.05		
Ordoñez et al. [50]	**Outcomes of anthropometry**						
	Fat mass (%)	CCT	38.9 ± 4	35 ± 3.8	<0.05	-	-
		CG	-	-	-	-	-
	Waist circumference (cm)	CCT	94.7 ± 3.3	91.5 ± 3.1	<0.05	-	-
		CG	-	-	-	-	-
	Waist-to hip ratio	CCT	1.12 ± 0.01	1 ± 0.01	<0.05	-	-
		CG	-	-	-	-	-
	**Lipid profile, hemodynamic and metabolic markers**						
	Plasma leptin levels (ng/mL)	CCT	54.2 ± 6.7	45.7 ± 6.1	<0.05	-	-
		CG	55.8 ± 6.9	55.4 ± 7	-	-	-
	**Outcome of cardiorespiratory function**						
	VO_2max_ (mL/kg/min)	CCT	20.2 ± 5.8	23.7 ± 6.3	<0.01	-	-
		CG	-	-	-	-	-
Ordonez et al. [51]	**Outcomes of anthropometry**						
	Fat mass (%)	CCT	38.9 ± 4	35 ± 3.8	<0.05	<0.05	-
		CG	37.7 ± 3.8	37.8 ± 3.9	-	-	-
	Waist circumference (cm)	CCT	94.7 ± 3.3	91.5 ± 3.1	<0.05	<0.05	-
		CG	93.5 ± 3.1	93.7 ± 3.2	-	-	-
	Waist-to hip ratio	CCT	1.12 ± 0.0006	1 ± 0.005	<0.05	<0.05	-
		CG	1.11 ± 0.005	1.11 ± 0.005	-	-	-
	**Lipid profile, hemodynamic and metabolic markers**						
	Tumors necrosis factor (pg/mL)	CCT	11.7 ± 1.6	9.2 ± 1.3	<0.05	<0.05	-
		CG	11.4 ± 1.5	11.5 ± 1.5	-	-	-
	Interleukin-6 (pg/mL)	CCT	8.2 ± 1.1	6.1 ± 0.9	<0.05	<0.05	-
		CG	8.2 ± 1.2	8.3 ± 1.3	-	-	-
	Fibrinogen (g/L)	CCT	3.72 ± 0.48	3.23 ± 0.41	<0.05	<0.05	-
		CG	3.74 ± 0.52	3.75 ± 0.52	-	-	-
	High sensitive C-reactive protein (mg/dL)	CCT	0.62 ± 0.11	0.53 ± 0.09	<0.05	<0.05	-
		CG	0.6 ± 0.1	0.61 ± 0.1	-	-	-
Ringenbach et al. [52]	**Outcome of cognition function**						
	Reaction time	CCT	0.784 ± 0.532	(Δ = −0.234 ± 0.354; Hedges’g = −0.42)	-	<0.05	-
		Voluntary CG	0.748 ± 0.691	(Δ = −0.02 ± 0.109; Hedges’g = −0.04)	-	-	-
		CG	0.716 ± 0.339	(Δ = −0.051 ± 0.234; Hedges’g = 0.09)	-	-	
	Set-shifting ability	CCT	4.65 ± 2.15	(Δ = 0.47 ± 1.77; Hedges’g = 0.19)	-	-	-
		Voluntary CG	4.25 ± 2.65	(Δ = 1.44 ± 2.39; Hedges’g = 0.57)	-	<0.05	-
		CG	4.36 ± 2.80	(Δ = 0 ± 0; Hedges’g = 0)	-	-	-
	Response inhibition	CCT	7.94 ± 5.83	(Δ = 1 ± 3.14; Hedges’g = 0.18)	-	-	<0.05 (vs. voluntary CG)
		Voluntary CG	10.31 ± 4.85	(Δ = −1.75 ± 3.57; Hedges’g = −0.32)	-	-	-
		CG	6.27 ± 5.57	(Δ = −1.36 ± 2.91; Hedges’g = −0.25)	-	-	-
	Semantic language fluency	CCT	16.19 ± 9.83	(Δ = 2.47 ± 4.45; Hedges’g = 0.25)	-	<0.05	-
		Voluntary CG	16.07 ± 10.27	(Δ = 2.2 ± 3.55; Hedges’g = 0.22)	-	<0.05	-
		CG	22.4 ± 9.45	(Δ = −1.7 ± 4.83; Hedges’g = −0.17)	-	-	-
Rosety-Rodriguez et al. [53]	**Outcomes of anthropometry**						
	Fat mass (%)	CCT	38.9 ± 4	35 ± 3.8	<0.05	-	-
		CG	37.7 ± 3.8	37.8 ± 3.9	-	-	-
	Waist circumference (cm)	CCT	94.7 ± 3.3	91.5 ± 3.1	<0.05	-	-
		CG	93.5 ± 3.1	93.7 ± 3.2	-	-	-
	**Lipid profile, hemodynamic and metabolic markers**						
	Interleukin-6 (pg/mL)	CCT	8.2 ± 1.1	6.1 ± 0.9	<0.05	-	-
		CG	8.1 ± 1.2	8.3 ± 1.3	-	-	-
	High-sensitive C-reactive protein (mg/dL)	CCT	0.62 ± 0.11	0.53 ± 0.09	<0.05	-	-
		CG	0.6 ± 0.1	0.61 ± 0.1	-	-	-
	**Outcome of cardiorespiratory function**						
	VO_2max_ (mL/kg/min)	CCT	20.2 ± 5.8	23.7 ± 6.3	<0.05	-	-
		CG	20.4 ± 5.5	20.6 ± 5.7	-	-	-

BMI—body mass index; CCT—continuous cardiorespiratory training; CG—control group; HDL—high-density lipoprotein; ICT—Interval cardiorespiratory training; LDL—low-density lipoprotein; M—mean; PPT—Purdue Pegboard Test; RLB—right, left and bimanual subtests; SD—standard deviation; VO_2max_—maximun oxygen: VO_2 peak_—peak oxygen uptake.
